# Psychological stress contributed to the development of low-grade fever in a patient with chronic fatigue syndrome: a case report

**DOI:** 10.1186/1751-0759-7-7

**Published:** 2013-03-08

**Authors:** Takakazu Oka, Yoshio Kanemitsu, Nobuyuki Sudo, Haruo Hayashi, Kae Oka

**Affiliations:** 1 Department of Psychosomatic Medicine, Graduate School of Medical Sciences, Kyushu University, Fukuoka 812-8582, Japan; 2 Section of Psychosomatic Medicine, Department of General Medicine, Fukuoka Dental College, Fukuoka, Japan; 3 Division of Psychosomatic Medicine, Department of Neurology, University of Occupational and Environmental Health, Iseigaoka 1-1, Yahatanishi-ku, Kitakyushu 807-8555, Japan; 4 Department of Pediatrics and Child Health, Kurume University School of Medicine, Asahi-machi 67, Kurume 830-0011, Japan

**Keywords:** Chronic fatigue syndrome, Stress-induced hyperthermia, Psychogenic fever, Stress interview, Cytokine

## Abstract

**Background:**

Low-grade fever is a common symptom in patients with chronic fatigue syndrome (CFS), but the mechanisms responsible for its development are poorly understood. We submit this case report that suggests that psychological stress contributes to low-grade fever in CFS.

**Case presentation:**

A 26-year-old female nurse with CFS was admitted to our hospital. She had been recording her axillary temperature regularly and found that it was especially high when she felt stress at work. To assess how psychological stress affects temperature and to investigate the possible mechanisms for this hyperthermia, we conducted a 60-minute stress interview and observed the changes in the following parameters: axillary temperature, fingertip temperature, systolic blood pressure, diastolic blood pressure, heart rate, plasma catecholamine levels, and serum levels of interleukin (IL)-1β and IL-6 (pyretic cytokines), tumor necrosis factor-α and IL-10 (antipyretic cytokines). The stress interview consisted of recalling and talking about stressful events. Her axillary temperature at baseline was 37.2°C, increasing to 38.2°C by the end of the interview. In contrast, her fingertip temperature decreased during the interview. Her heart rate, systolic and diastolic blood pressures, and plasma levels of noradrenaline and adrenaline increased during the interview; there were no significant changes in either pyretic or antipyretic cytokines during or after the interview.

**Conclusions:**

A stress interview induced a 1.0°C increase in axillary temperature in a CFS patient. Negative emotion-associated sympathetic activation, rather than pyretic cytokine production, contributed to the increase in temperature induced by the stress interview. This suggests that psychological stress may contribute to the development or the exacerbation of low-grade fever in some CFS patients.

## Background

Patients with chronic fatigue syndrome (CFS) frequently exhibit low-grade fevers [1], but the mechanisms responsible for this phenomenon are poorly understood. We hypothesize that psychological stress contributes to the development or the exacerbation of low-grade fever in these patients. Some CFS patients experience workday hyperthermia even at sedentary jobs, exhibiting higher axillary temperatures on working days compared with holidays [2]. Many studies have demonstrated that psychological stress affects core body temperature in laboratory animals, with acute stress inducing transient hyperthermia [3,4] and repeated, chronic stress inducing persistent low-grade hyperthermia [5,6] and facilitating a hyperthermic response to a novel stressor [7]. For a review of this topic, please see Oka et al. (2001), as well as Oka and Oka (2012) [8,9].

So far, however, there have been no reports demonstrating that psychological stress can directly affect the body temperature of CFS patients. We present a patient with CFS in whom a stress interview increased axillary tem-perature by up to 1.0°C. To our knowledge, this is the first case report demonstrating that a stress interview actually increased body temperature in a CFS patient. Furthermore, this is also the first report to document changes in pyretic and antipyretic cytokines during a stress interview in a CFS patient.

## Case presentation

Patient: A 26-year-old woman.

Chief complaints: Low-grade fever, general fatigue, arthralgias, myalgias, photophobia, and difficulty concen-trating.

Family history: No family history of CFS or depressive disorders.

Past medical history: Necrotizing lymphadenitis at the age of 20 years. Endometriosis diagnosed at the age of 20 years, with oral contraceptive use since that time.

### History of present illness

The patient visited an outside hospital at the age of 20 years, when she was a nursing student, with the complaint of fever, around 38°C, and general fatigue. She was diagnosed with necrotizing lymphadenitis and treated accordingly. Although her physician considered her disease cured, her low-grade fever and fatigue persisted and strong malaise, particularly post-exertional, caused her to abandon her studies. At the age of 22, she was hospitalized in our department and diagnosed with CFS after a thorough medical examination. Active psychiatric and medical diseases were excluded and she fulfilled both the 1988 working definition of CFS given by the Centers for Disease Control and Prevention (CDC) [10] and the CDC’s 1994 Fukuda definition of CFS [11]. Her condition gradually improved with antidepressant therapy. As her fatigue improved to 1–2 on a numerical rating scale (NRS), with 10 representing the worst fatigue in her experience and 0 representing no fatigue, she began another nursing program. She graduated at the age of 26 and began work as a nurse in an intensive care unit, but 4 months later she stopped work due to exacerbation of her fatigue and low-grade fevers. Her symptoms persisted despite 2 months of rest, and she was referred again to our department where she was hospitalized. On admission, she complained of persistent fatigue and fevers. She scored the severity of her fatigue as 6–8 and demonstrated an injected pharynx; tenderness was present in her right cervical lymph nodes. Again, she satisfied both CDC definitions of CFS.

She had been checking her own temperature and noted that, before hospitalization, her axillary temperature was 37.5–38.0°C in the afternoon; antipyretic medications such as acetaminophen failed to reduce this low-grade fever. She noticed that her axillary temperature reached 38.5°C at work, especially when she was in a situation of psychological stress such as a meeting with her head nurse. After hospitalization, her axillary temperature decreased to 37.0–37.5°C in the afternoon. On the 8th day of hospi-talization, we conducted a 1-hour stress interview to assess how psychological stress affected her body temperature, and the possible mechanisms for this effect. We investigated the involvement of sympathetic activation and of the pyretic cytokines interleukin (IL)-1β and IL-6 and the antipyretic cytokines IL-10 and tumor necrosis factor (TNF)-α [12].

### Stress interview

At 9:00 AM on the day of the interview, a soft cannula for blood collection was inserted in a vein in the patient’s right forearm. The 1-hour stress interview began at 2:00 PM; this time was chosen for the interview because her axillary temperature had shown little change between 2:00 PM and 5:00 PM since admission. At 1:30 PM, she entered an interview room which was maintained at 23°C. This room was familiar to her because she had already visited it several times for psychotherapy. An electronic manometer cuff was placed around her left upper arm and thermal probes were placed in her left ear and on her left index fingertip. Her axillary body temperature, systolic blood pressure (SBP), diastolic blood pressure (DBP), and heart rate (HR) were measured at fixed time intervals. During the interview, we asked her to tell us about difficulties in her life. She was also informed that we would stop the interview at any point, if she so desired.

After the interview was completed, she was asked to stay in the room for a further 30 minutes, for a total of 90 min from the start of the interview. She then returned to her own room. Tympanic membrane and fingertip temperatures were monitored once every minute during her stay in the interview room. Her axillary temperature, SBP, DBP, and HR were measured every 15 minutes, from 0 min to 60 min after the start of the interview and again at 90 min, 120 min, and 180 min after starting the interview. Blood samples were collected at baseline (−15 min) and at 30 min, 60 min, 120 min, and 180 minutes. Blood samples were stored at −80°C until the catecholamine and cytokine measurements were performed.

The following is a transcription of the stress interview questions and answers.

Doctor (Dr): Could you tell me how you felt when you had to leave the first nursing school? (0–15 min)

Patient (Pt): My friends graduated from school and became nurses but I couldn’t (starts to sob). I envied my friends. And I felt sorry for my parents.

Dr: Could you tell me more detail about how you felt for your parents? (16–30 min).

Pt: My mother came to the hospital to see me every other day but I didn’t tell her anything about how I felt. When I had to be absent from school for hospitalization, I told my teacher about my diagnosis. She said ironically, “chronic fatigue? It’s a good name. I always feel fatigue but I have to work.” She was a nurse, of course. I couldn’t believe she would say that. At that time, I thought nobody could understand me, including my mother. So I didn’t tell her anything.

Dr: You didn’t give up your dream to be a nurse and went to another school, right? How was your second school life and the hospital where you worked? (31–45 min)

Pt: I thought I couldn’t fail this time. So I studied feverishly but I forgot many things the next day. I had to set my goals very low.

Dr: What was the hardest time in your life? Could you tell me how you thought about this disease? (46–60 min)

Pt: When I left the first school, because I had enough credits to graduate. I don’t think this disease is curable.

During the interview, the patient talked with tears in her eyes about how hard her situation had been. She reported gradually feeling hot as the interview proceeded, but never felt cold.

### Measures

SBP, DBP, and HR were recorded by an electronic sphygmomanometer (Nico PS501, Parama-Tech Co. Ltd., Fukuoka, Japan). The tympanic membrane temperature was monitored with a DBLT-1/WL thermal probe (Ymatic Ltd., Tokyo, Japan), the fingertip temperature was monitored using a ProComp Infiniti probe (Thought Technology, Ltd., Montreal, Canada), and the axillary temperature was measured with a MC-440 probe (Terumo Medical Corporation, Tokyo, Japan). Plasma catecholamine levels were measured by high-performance liquid chromatography. Serum cytokine levels were measured by a quantitative sandwich enzyme immunoassay technique using Quantikine HS immunoassay kits (R&D Systems, Inc., Minneapolis, MN, USA) for IL-1β IL-6, and TNF-α. IL-10 was assayed using a human IL-10 Enzyme Amplified Sensitivity Immunoassay kit (Bioscience Europe S.A., Brussels, Belgium). The minimum detectable concentrations of IL-1β, IL-6, TNF-α, and IL-10 were 0.125 pg/mL, 0.156 pg/mL, 0.5 pg/mL, and 2 pg/mL, respectively. Subjective severity of fatigue was scored on a NRS, with 10 representing the worst fatigue possible and 0 representing no fatigue.

### Changes in body temperature and fatigue levels

**Figure 1:**
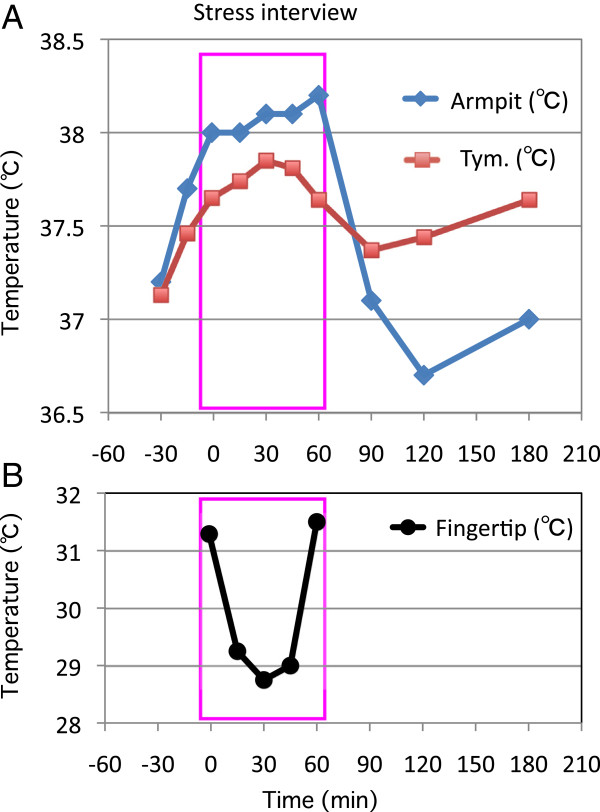
**Temperature changes with stress interview.** Changes in axillary (armpit) and tympanic membrane (tym.) temperatures (**A**) and fingertip temperature (**B**) during and after a 60-minute stress interview.

The patient’s axillary temperature was 37.1°C at 9 AM, and 37.2°C when she entered the interview room at 1:30 PM. It gradually increased, reaching a maximum of 38.2°C at the end of the 60-min interview, then decreased to the pre-interview level of 37.1°C, 30 min after completion of the interview (90 min from the interview’s start). The tympanic membrane temperature also increased, but the magnitude of this increase was less than that of the axillary temperature. Her tympanic membrane temperature was 37.1°C when she entered the interview room, reached a maximum of 37.9°C, 30 min after starting the interview, and gradually decreased to 37.4°C, 30 min after the end of the interview (90 min from the interview’s start). Her fingertip temperature was 31.3°C before the interview, 28.7°C–29.2°C during the interview, and 31.5°C at the end of the interview (Figure 1). The severity of her fatigue on the NRS was 7 at 9 AM and 6 at 1:30 PM. It had increased to a level of 9 by the end of the interview and decreased to 6 at 5 PM (180 min from the interview’s start).

In comparison, the patient’s axillary temperature at noon, 2 PM, and 4 PM were 37.4°C, 36.8C, and 37.3C, respectively, two days after the stress interview when she didn’t feel any stress. The patient remained seated during the interview. The observed increase in axillary and tympanic membrane temperatures during the interview were therefore not due to diurnal body temperature changes or changes in activity.

### Changes in cardiovascular parameters, peripheral cytokines, and catecholamine levels

Plasma levels of noradrenaline and adrenaline increased during the stress interview at 30 min and 60 min, and returned to their pre-interview values 1 hour after the interview (120 min). The patient’s SBP, DBP, and HR were also higher during the interview than at 9 AM, than during the pre-interview period (−15 min), and than 2 hours after the interview (180 min). In contrast, neither pyretic nor antipyretic cytokines showed significant changes during the interview. Rather, IL-6 and TNF-α were slightly suppressed during the interview and 1 hour afterwards (IL-6 suppressed at 30 min and 60 min; TNF-α suppressed at 60 min and 120 min), and returned to pre-interview levels 2 hours after the interview (180 min). IL-10 remained under the minimum detectable level throughout the observation period (Table 1).

**Table 1:** Changes in cardiovascular parameters, cytokines, and catecholamines during and after a 60-min stress interview

	**9 am**	**pre**	**30 min**	**60 min**	**120 min**	**180 min**
IL-1β (pg/ml)	0.39	0.29	0.3	0.33	0.28	0.37
IL-6 (pg/ml)	2.9	3.1	1.8	1.9	3.6	3.3
TNF-α (pg/ml)	1.5	1.3	1.4	1.1	1	1.5
IL-10 (pg/ml)	<2	<2	<2	<2	<2	<2
A (pg/ml)		36	65	59	36	
NA (pg/ml)		298	409	431	285	
DA (pg/ml)		9	10	14	<5	
SBP (mmHg)	100	116	126	121	122	106
DBP (mmHg)	66	79	97	93	77	75
HR (mmHg)	72	92	103	102	83	86

### Clinical course

**Figure 2:**
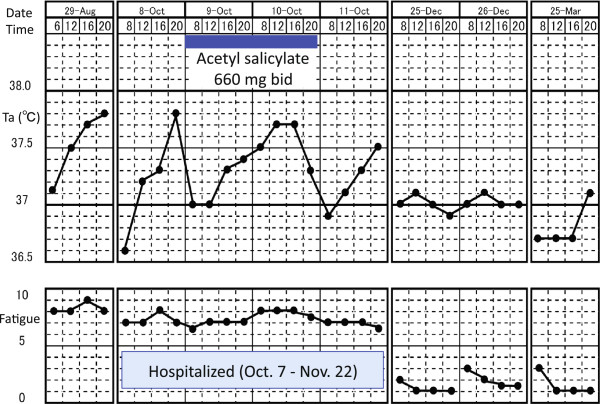
**Clinical course.** Axillary temperature was measured 4 times daily: at 8:00AM; 12:00PM; 4:00PM; and 8PM. Fatigue was scored by using a numerical rating scale with 10 representing maximum fatigue and 0 representing no fatigue.

During her hospitalization, the patient was treated with combination biomedical and psychosocial therapy. She was housed in a calm inpatient setting, amenable to self-reflection. After a period of bedrest, she was asked to take the time to be mindful, fully tasting her food and taking relaxed walks, hearing the birds, watching the trees and flowers, and feeling the wind. The aim was to facilitate the restoration of interoceptive awareness, previously suppressed by her extreme exertions to overcome fatigue. We also wanted to replace her coping mechanisms, which led to exacerbation of her fatigue. Her treatment also involved (1) pharmacotherapy including hochuekkito (a Japanese herbal medicine), mirtazapine, and meco-balamin; (2) cognitive and behavioral intervention focusing on noticing thresholds that worsen her fatigue and changing work habits and cognition patterns when tired; (3) relaxation training, including group sessions on autogenic training, followed by self-practice; (4) emotional disclosure during supportive psychotherapy; (5) graded exercise therapy; (6) reconstructing her relationship with her mother; and (7) making environmental arrangements at her workplace. Her low-grade fever and fatigue gradually improved and she returned to work (Figure 2).

## Conclusions

This study demonstrates that a stress interview may increase the axillary and tympanic membrane temperatures of a patient with CFS. Both temperatures began to increase 30 min before starting the interview; this may have been caused by anticipatory stress, as has been shown in animal models [13]. This study also demonstrates that the pyretic and antipyretic cytokines are not involved in stress interview-induced hyperthermia.

Activation of the sympathetic nervous system is known to increase core body temperature by increasing thermogenesis, including non-shivering thermogenesis in brown adipose tissue, and by decreasing heat loss with peripheral vasoconstriction [8,9]. Considering that the increase in axillary and tympanic membrane temperatures and the decrease in finger temperature were associated with an increase in blood catecholamine levels, sympathetic activation could play an important role in these temperature changes.

This study showed that a 1-hour stress interview was able to induce a robust increase in axillary temperature of up to 1.0°C (to 38.2°C). The psychological stress-induced hyperthermic response exists not only in this patient but also in healthy human subjects. However, the available studies suggest that the magnitude of the response in our patient is much larger than that typically seen [14,15]. For example, the mean axillary temperature of psychiatry residents between 26 and 33 years of age, taken 10 to 15 minutes before an examination (37.0°C), is 0.6°C higher than the axillary temperature taken 2 to 3 weeks later in the same room (36.4°C), when the subjects are in a calm situation [14]. Another study showed that the oral temperature measured in students between 18 and 27 years of age immediately before an examination (37.4°C) is 0.18°C higher than the oral temperature taken 3 days after the examination (37.22°C), at the same hour of the day [15]. Animal studies have demonstrated that sympathetic thermogenic action is enhanced in a repeated/chronic stress situation compared with a non-stressful situation [16]. It is possible that our patient’s difficult life situation acted as a chronic stressor, leading her to exhibit a robust increase in axillary temperature as an acute stress exposure.

In this patient, we found that the magnitude of the stress-induced axillary-temperature increase was larger than the tympanic-membrane temperature increase. Animal studies have demonstrated that non-shivering thermogenesis, taking place in brown adipose tissue, plays a crucial role in the development of stress-induced hyperthermia [4]. In human adults, brown adipose tissue is densely distributed in the paracervical and supraclavicular regions [17,18]. If a stress interview activates brown adipose thermogenesis, resulting in hyperthermia, it is reasonable to presume that the increase in temperature will be more evident in the axilla, which is at closer proximity to brown adipose tissue than the tympanic membrane. This study suggests that the axillary temperature may be a better index for assessing the effect of stress on the body temperature of CFS patients.

The results described in this case report suggest that psychological stress contributes to the development or exacerbation of low-grade fever in some CFS patients, possibly via sympathetic activation. Peripheral cytokines may not be involved in this process.

## Consent

Written informed consent was obtained from the patient for publication of this case report and any accompanying images. A copy of the written consent is available for review by the Editor-in-Chief of this journal.

## Abbreviations

A: Adrenaline; CFS: Chronic fatigue syndrome; DA: Dopamine; DBP: Diastolic blood pressure; Dr: Doctor; HR: Heart rate; IL-1β: Interleukin-1β; IL-6: Interleukin-6; IL-10: Interleukin-10; Min: Minutes; NA: Noradrenaline; Pt: Patient; SBP: Systolic blood pressure; TNF-α: Tumor necrosis factor-α.

## Competing interests

The authors declare that they have no competing interests.

## Authors’ contributions

TO designed the study protocol, treated the patient, analyzed the data, and drafted the manuscript. YK, HH, and KO helped with assays and advised on data analysis. NS looked over the study. All authors read and approved the final manuscript.
